# Using Geographical Convergence of Obesity, Cardiovascular Disease, and Type 2 Diabetes at the Neighborhood Level to Inform Policy and Practice

**DOI:** 10.5888/pcd14.170170

**Published:** 2017-10-12

**Authors:** Kayla Smurthwaite, Nasser Bagheri

**Affiliations:** 1 Australian National University, Canberra, Australia

## Abstract

**Introduction:**

Chronic diseases are increasing across the world. Examination of local geographic variation in chronic disease patterns can enable policy makers to identify inequalities in health outcomes and tailor effective interventions to communities at higher risk. Our study aimed to determine the geographic variation of obesity, cardiovascular disease (CVD), and type 2 diabetes, using general practice clinical data. Further objectives included identifying regions of significantly high and low clusters of these conditions and assessing their association with sociodemographic characteristics.

**Methods:**

A cross-sectional approach was used to determine the prevalence of obesity, CVD, and type 2 diabetes in western Adelaide, Australia. The Getis-Ord Gi* method was used to identify significant hot spots of the conditions. Additionally, we used the Pearson correlation test to determine the association between disease clusters and risk factors, including socioeconomic status (SES), smoking history, and alcohol consumption.

**Results:**

The spatial distribution of obesity, CVD, and type 2 diabetes varied across communities. Hot spots of these conditions converged in 3 locations across western Adelaide. An inverse relationship was observed between area-level prevalence of CVD, obesity, and type 2 diabetes with SES.

**Conclusion:**

Identification of significant disease clusters can help policy makers to target prevention strategies at the right people, in the right location. The approach taken in our study can be applied to identify clusters of other chronic diseases across the world, wherever researchers have access to clinical data.

**Editor’s Note:** This article is the winner of *PCD*‘s 2017 Student Research Paper Contest in the Undergraduate category.

## Introduction

The global prevalence of obesity is a major threat to public health because of its steep increase in recent years (1,2). This trend is of international concern, with over 13% of men and 21% of women in the world classified as obese according to their body mass index (BMI) (1). Although the financial burden of high BMI raises concerns about the effectiveness of intervention strategies (3–5), increasingly more attention is placed on the role of obesity in the development of other chronic diseases (1). The relationship between obesity, cardiovascular disease (CVD), and type 2 diabetes mellitus is well documented, with high BMI associated with the development of atherosclerosis, hypertension, and insulin resistance (6–9). Because obesity is a clear risk factor of chronic disease, questions are raised about whether obese populations have higher rates of CVD and type 2 diabetes.

The reported interplay of sociodemographic characteristics and lifestyle factors in the development of adulthood obesity supports the notion that high BMI is not randomly distributed within a population (3,10). Therefore, the implementation of intervention programs that target all individuals within a population are limited in their capacity to create change. Intervention strategies need to be tailored to communities where clusters of obesity, CVD, and type 2 diabetes exist. Examination of local geographic variation and the identification of the hot spots is a novel approach to inform policy and practice about inequalities in health outcomes.

The primary objective of our study was to determine the geographic variation of obesity, CVD, and type 2 diabetes in an Australian community, using general practice clinical data. Secondary objectives included the identification of regions of significantly high and low clusters of these conditions and the determination of their relationship with sociodemographic characteristics.

## Methods

A clinical data set of de-identified patient records (n = 84,387) from 2010 through 2014 was acquired from 16 general practices across western Adelaide, South Australia. Obesity was defined as a BMI of 30.0 or higher, as calculated by clinical measurements of an individual’s weight and height (kg/m^2^). CVD was defined as having at least 1 of the following 5 CVD events: carotid stenosis, chronic heart disease, heart failure (chronic and acute), myocardial infarction, or peripheral vascular disease. Active type 2 diabetes was defined by prior diagnosis from a medical practitioner. The study obtained ethics approval from the Australian National University Human Ethics Committee (protocol 2014/174).

Data analysis was restricted to individuals aged 35 to 74 years. Active patients (individuals who had visited their general practitioner at least 3 times between 2012 and 2014) with complete data on sociodemographic characteristics and geographic information were included in the individual-level analysis (n = 20,594). After exclusion of individuals residing outside of western Adelaide, patients’ medical records were geo-linked to Australian Bureau of Statistics (ABS) Statistical Area Level 1 (SA1) regions (mean, 400 individuals per SA1) (11). Active patients across 490 SA1 regions (n = 17,716; mean, 36 patients per SA1) were included in the population-level analysis. Only SA1 regions with 5 or more patients were included to preserve patient privacy.

### Descriptive analysis

Mean BMI and frequency of CVD and type 2 diabetes diagnosis were determined for sex, age category, and SES through use of the Stata software (version 14; StataCorp LP). SES was classified into tertiles based on ABS Socioeconomic Indexes for Areas (SEIFA) data, including low socioeconomic, moderate socioeconomic, and high socioeconomic regions (12). Mean BMI and disease frequency was further calculated for each discrete BMI category, including the underweight class (<18.5), normal class (18.5–24.9), overweight class (25.0–29.9) and obese class (≥30.0). Additionally, mean BMI and disease frequency was determined for tobacco smoking status, frequency of alcohol consumption, and total cholesterol level. Using definitions from the Metadata Online Registry (13), individuals were classified as having smoked tobacco throughout their life or as not having smoked. Individuals who consumed alcohol at least once in the past year were identified as alcohol consumers and those who had not consumed alcohol in the past year were identified as not alcohol consumers (13). Cholesterol levels were classified as either normal or high: normal cholesterol was defined as less than 5.5 mol/L and high cholesterol was defined 5.5 mol/L or higher (14). For all risk factors, individuals with incomplete records were excluded from the descriptive analysis. The percentage of individuals with CVD and type 2 diabetes in each subpopulation was calculated by direct standardization to allow for within-group and between-group comparisons. The statistical significance of the difference in BMI and disease prevalence between each subpopulation was further calculated using the non-parametric Kruskal–Wallis test.

### Spatial analysis

Mean BMI and percentage of individuals diagnosed with CVD and type 2 diabetes were aggregated at the SA1 level for western Adelaide. The regional variation was mapped across western Adelaide communities for continuous values of BMI, CVD, and type 2 diabetes diagnosis. Values were categorized into 4 groups using the Jenks natural breaks classification method (separation of data based on naturally occurring groups, determined to be the best arrangement of data) (15). A similar technique was used to map the geospatial variation of SES in western Adelaide, although SA1 regions were instead categorized into 3 groups based on the ABS SEIFA tertiles.

Local spatial clusters at the SA1 level with obesity, CVD, and type 2 diabetes were examined using the Getis-Ord Gi* technique (16,17). This tool compares the local sum of, for example, obesity values (the sum of obesity values of the targeted SA1 area and its neighboring SA1s) to the sum of obesity values of all SA1s within the study area. A significant, positive *z* score indicates a local high-rate cluster (hot spot). Hot spots are detected when an SA1 with high rates of disease is surrounded by SA1s that also have high rates of disease; the observed local sum of disease is higher than the expected local sum, and the difference is too large to be the result of chance alone. Similarly, a significant, negative *z* score indicates a local low-rate cluster (cold spot), where an SA1 with low rates is surrounded by SA1s with low rates (16,17). Significant hot spot and cold spot clusters were visualized in the western Adelaide area to highlight communities with high rates and low rates of obesity, CVD, and type 2 diabetes.

Resulting visual representations of the spatial distribution of obesity, CVD, and type 2 diabetes promoted comparison of disease hot spots and cold spots, allowing conclusions to be made about the convergence of the 3 conditions. Pearson correlation statistics were used to determine the global relationship between SES and the 3 conditions, with further comparisons made between the prevalence of CVD and type 2 diabetes. For the spatial analysis, we used ArcGIS software (version 10.4, Esri).

## Results

### Descriptive statistics

The prevalence of obesity in the sample population was 43.2% (Table 1). Mean BMI across sex, age category, and SES was constant, with total variation at most 1.4 kg/m^2^ between high SES and low SES. Men had a significantly higher BMI than women (*P* < .001) and increasing age had a significant, positive relationship with increasing BMI (*P* < .001). This trend was further seen for CVD and type 2 diabetes diagnosis, with men reporting a significantly higher diagnosis rate than women (*P* < .001 for each). CVD prevalence was 3 times higher in men than in women, where 9.1% of men reported at least 1 cardiovascular event throughout their life. Type 2 diabetes diagnosis rates were 3 percentage points higher in men than in women.

**Table 1:** Distribution of Mean Body Mass Index (BMI)^a^, Type 2 Diabetes Diagnosis, and Cardiovascular Disease (CVD) Event Diagnosis^b^ in Individuals Across Demographic Characteristics in General Practice Clinical Data (N = 20,594), Western Adelaide, South Australia

Demographic Characteristic	No. (%)	Mean BMI	Type 2 Diabetes Diagnosis, No. (%)	CVD Event, No. (%)
**Sex**				
Male	9,190 (44.6)	30.0	1,154 (12.6)	839 (9.1)
Female	11,404 (55.4)	29.9	1,054 (9.2)	366 (3.2)
**Age, y**				
35–44	3,884 (18.9)	29.5	105 (2.7)	21 (0.5)
45–54	6,183 (30.0)	29.9	359 (5.8)	133 (2.2)
55–64	5,531 (26.9)	30.2	732 (13.2)	474 (8.6)
65–74	4,996 (24.3)	30.1	696 (13.9)	1,013 (20.3)
**Socioeconomic status^c^**				
Low	7,065 (34.3)	30.6	843 (11.9)	495 (7.0)
Moderate	6,710 (32.6)	30.1	750 (11.2)	373 (5.6)
High	6,819 (33.1)	29.2	616 (9.0)	377 (5.5)

a Calculated by clinical measurements of an individual’s weight in kilograms and height in meters squared.

b At least 1 of 5 CVD events: carotid stenosis, chronic heart disease, heart failure (chronic and acute), myocardial infarction, and peripheral vascular disease.

c Classified into tertiles based on Australian Bureau of Statistics Socioeconomic Indexes for Areas data (12).

The prevalence of CVD events and type 2 diabetes also had a significant, positive association with increasing age (*P* < .001 for each), with adults aged 65 to 74 years reporting the highest rate of diagnosis. In comparisons between the age groups of 35 to 44 and 65 to 74 years, the prevalence of CVD events among older adults was 40 times higher than that in younger adults, and the occurrence of type 2 diabetes diagnosis was 5 times higher in adults aged 65 to 74 years (Table 1).

Differences in disease prevalence related to SES were smaller than those associated with sex and age (Table 1). Individuals with high SES had lower diagnosis rates of CVD or type 2 diabetes than did individuals with a low or moderate SES. This inverse relationship indicates that even individuals with a moderate SES have a lower prevalence of all conditions than those in the lowest tertile. However, differences were only at most 1.4% lower across the sample population for CVD events.

The percentage of individuals diagnosed with type 2 diabetes and CVD had a significant, positive association with increasing BMI (*P* < .001 for each). Obese individuals had a higher rate of type 2 diabetes (4.4 times higher) and CVD events (2.1 times higher) than those in the normal BMI range (Table 2).

**Table 2:** Distribution of Mean Body Mass Index (BMI)^a^, Type 2 Diabetes Diagnosis, and Cardiovascular Disease (CVD) Event Diagnosis^b^ in Individuals Across Related Risk Factors in General Practice Clinical Data (N = 20,594), Western Adelaide, South Australia

Risk Factor	No.^c^ (%)	Mean BMI	Type 2 Diabetes Diagnosis, No. (%)	CVD Event, No. (%)
**BMI category**				
Underweight (<18.5)	226 (1.1)	17.4	5 (2.2)	6 (2.7)
Normal (18.5–24.9)	4,275 (20.8)	22.6	155 (3.6)	148 (3.5)
Overweight (25.0–29.9)	7,198 (34.4)	27.5	639 (8.9)	419 (5.8)
Obese (≥30.0)	8,895 (43.2)	35.8	1,410 (15.9)	633 (7.1)
**Cholesterol level**				
Normal (<5.5 mol/L)	12,639 (61.4)	30.2	1,882 (14.9)	1,063 (8.4)
High (≥5.5 mol/L)	6,762 (32.8)	29.5	312 (4.6)	127 (1.9)
**Smoking status**				
Has smoked throughout life	9,001 (43.7)	30.1	1,028 (11.4)	753 (8.4)
Never smoked	9,913 (48.1)	29.9	1,061 (10.7)	399 (4.0)
**Alcohol consumption**				
Consumes alcohol^d^	5,180 (25.2)	29.6	571 (11.0)	170 (3.3)
Never consumes alcohol	1,514 (7.4)	30.8	274 (18.1)	126 (8.3)

a Calculated by clinical measurements of an individual’s weight in kilograms and height in meters squared.

b At least 1 of 5 CVD events: carotid stenosis, chronic heart disease, heart failure (chronic and acute), myocardial infarction, and peripheral vascular disease.

c Numbers may not add to total N because of missing data.

d Consumed alcohol at least once in the past year.

Individuals with high total cholesterol levels did not have a higher prevalence of type 2 diabetes or CVD events than individuals with normal cholesterol levels (Table 2). We found an inverse relationship between cholesterol level and obesity, CVD, and type 2 diabetes. The highest percentage-point difference was for type 2 diabetes diagnosis, where individuals with normal cholesterol levels had a 10 percentage-point higher prevalence of type 2 diabetes than those with high cholesterol levels. However, data on cholesterol level were missing for 1,193 individuals, which may have changed the associations between cholesterol level and disease prevalence.

Individuals who reported a history of smoking had a higher prevalence of type 2 diabetes or CVD (*P* < .001 for each). In contrast to the results on smoking, we found an overall inverse relationship between alcohol consumption and disease occurrence. However, this relationship was not significant for CVD (*P* = .50) or type 2 diabetes diagnosis (*P* = .62). Although the association was not significant, 67.5% of the total sample population did not have complete reports of their alcohol consumption. This could have changed the relationship between alcohol use and type 2 diabetes and CVD prevalence.

### Spatial analysis

**Figure 1:**
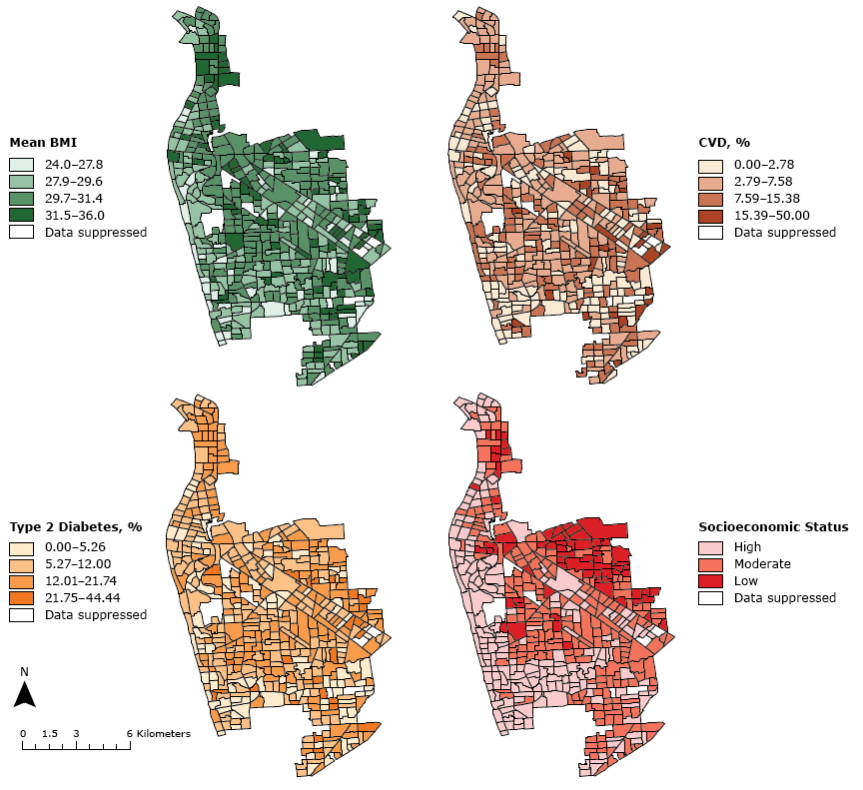
Regional variation of mean body mass index (BMI) (as calculated by clinical measurements of an individual’s weight and height [kg/m^2^]), cardiovascular disease event (CVD) diagnosis (%), type 2 diabetes diagnosis (%), and socioeconomic status, by Australian Bureau of Statistics Statistical Area Level 1 region, in western Adelaide, South Australia.

The regional distribution of BMI, CVD diagnosis (%), and type 2 diabetes (%) across western Adelaide indicated that the prevalence of the conditions varied across SA1 regions (Figure 1). Thematic maps (choropleth maps) show that the mean BMI of SA1 regions in western Adelaide was largely skewed toward the obese BMI class. Across the 490 SA1 regions, the lowest and highest reported mean BMIs were 24.0 and 36.0, respectively. The population-level rate of CVD was higher than that of type 2 diabetes.

The western coastline of western Adelaide has the lowest levels of obesity. This area is similar to the high-SES SA1 regions. We found a significant, inverse correlation between SES and mean BMI (−0.278) through Pearson correlation statistics. A similar relationship was shown for SES and CVD (−0.126), and SES and type 2 diabetes (−0.187). Disease patterns of CVD and type 2 diabetes had a significant, positive relationship (0.224).

**Figure 2:**
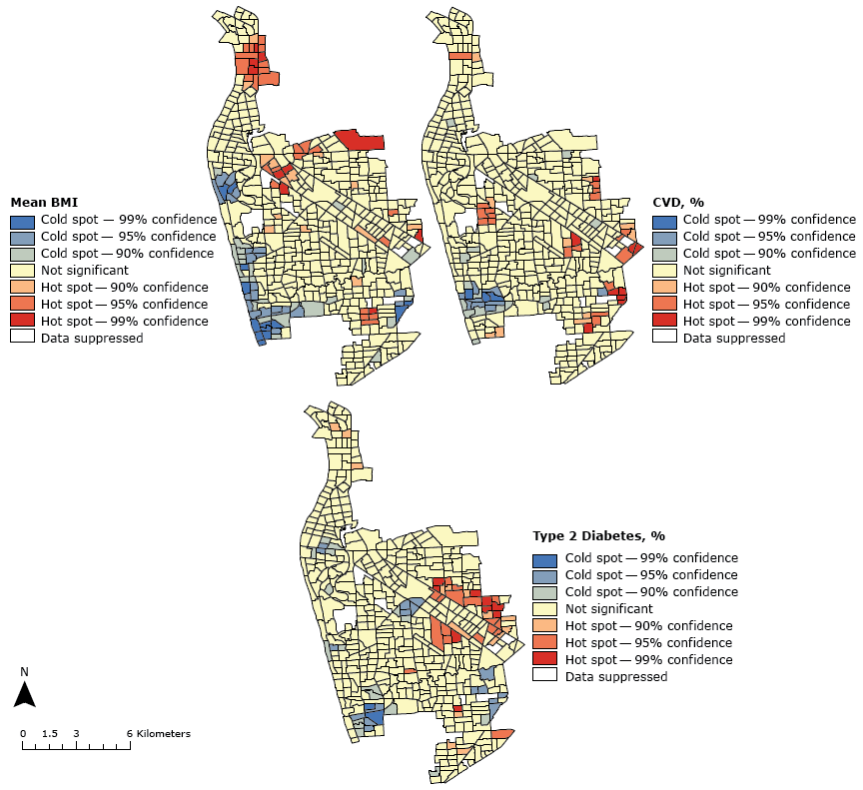
Hot spots and cold spots of mean body mass index (BMI) (as calculated by clinical measurements of an individual’s weight and height [kg/m^2^]), cardiovascular disease (CVD) event diagnosis (%), and type 2 diabetes diagnosis (%), by Australian Bureau of Statistics Statistical Area Level 1 regions, western Adelaide, South Australia.

Getis-Ord Gi* calculations determined regions across western Adelaide where the prevalence of mean BMI, CVD, and type 2 diabetes was significantly higher than other regions (Figure 2). We found 48 hot spots for BMI; they were primarily in the northern and eastern regions of western Adelaide. High-BMI cold spots were on the western coastline (Figure 2) and were associated with higher SES SA1 regions (Figure 1).

The spatial distribution of CVD events and type 2 diabetes also related to northern and central-eastern SA1 regions in western Adelaide. For CVD, 2 hot spots were found in the northern region and 26 hot spots toward the eastern region (Figure 2). For type 2 diabetes, we found a clustering of 32 hot spots in the central-eastern part of the study area. Furthermore, we observed geographical convergence for cold spots of high BMI, CVD, and type 2 diabetes in the southwestern region of western Adelaide (Figure 2), where SES is high (Figure 1).

## Discussion

Through combining geospatial analysis and general practice clinical data, our study aimed to determine the spatial variation of obesity, CVD, and type 2 diabetes in western Adelaide communities. Descriptive analysis of the study population revealed a positive association between high BMI and diagnosed CVD and type 2 diabetes. Identification of disease hot spots further showed geographic convergence of the 3 chronic diseases.

As supported by data from the Australian Institute of Health and Welfare (18,19), increasing age was positively associated with the increasing proportion of CVD and type 2 diabetes diagnoses. Mapping of mean BMI across demographic characteristics also aligned with trends found in literature (14,20), indicating that the sample used for the analysis was representative of other Australian communities. This is further established in the relationship between physiologic and lifestyle risk factors determined throughout the individual-level study, where increased BMI was associated with higher disease prevalence.

To date, population health researchers in Australia have not investigated the geographic variation of obesity, CVD, and type 2 diabetes at the neighborhood level. However, a study by Paquet et al (21) determined the clustering of biological risk factors related to the development of cardiometabolic diseases. The research emphasized the importance of using medical data collected by trained clinicians in determining the geographic spread of cardiometabolic outcomes and further outlined how clustering differs in relation to the geographic level analyzed (21). The level of spatial analysis completed by Paquet et al (21) was limited relative to our study. The intra-class correlation analysis was insufficient to determine the geographic hot spots and cold spots of cardiometabolic outcomes, investigating only the difference in the level of clustering of the risk factors (21). Thus, our study responds to a gap in the research of the spatial distribution of obesity, CVD, and type 2 diabetes across communities in Australia.

The overall aims of our study related to the population level and centered on using methods that would result in information that could be used to guide health policy and program implementation in the community. We found obesity, CVD, and type 2 diabetes hot spots in the northern and central-eastern SA1 regions. These hot spots could be a priority for policy interventions. Because these hot spots were further associated with populations of a low SES, there are further implications for the equality of health care access in the western Adelaide community. The problem of health care disparities may need to be more effectively monitored through longitudinal surveillance and related health care policies.

Our study has limitations. Use of clinical data is favored by Australian guidelines in assessing the prevalence of diseases in communities (20). Despite this, selection of study participants from local general practice records creates questions of bias. Although Australian data indicate that 85% of individuals visit their local general practitioner annually (22), the generalizability of our study is limited because individuals who visit their doctor are those who are sick and require medical attention. This selection bias may account for the larger prevalence of obesity, CVD, and type 2 diabetes shown in our study, in comparison to findings reported by the ABS (14). A further limitation in the generalizability of our study is its cross-sectional design. Because the analysis did not longitudinally follow participants, if individuals move to a different location, the identified disease hot spots and cold spots may not continue to represent the frequency of obesity, CVD, and type 2 diabetes in the SA1 regions.

In line with emerging recommendations from the World Health Organization, waist circumference, in addition to BMI, should be used to diagnose obesity in individuals (23). Therefore, we could improve our study approach by changing how we measure obesity. Because waist circumference measurements were not accurately reported in the general practice data used for our study, we could not use these data. Areas of future research could also include a qualitative study to determine the sociodemographic characteristics and lifestyle risk factors related to obesity, CVD, and type 2 diabetes. Through use of the South Australian Monitoring and Surveillance System (24), our approach could be extended to analyze the differences between identified hot spots and cold spots within the community, providing further evidence for changes to government policies and programs. In addition, a quantitative investigation into the access and use of primary care in western Adelaide could be developed to determine the effect of health care disparities on the spatial distribution of obesity, CVD, and type 2 diabetes. Further analysis of community disease profiles at the small-area level would allow more conclusions to be made about the most effective aspects of prevention and intervention programs and could be seen as an improvement to the new approach presented here.

Combining geospatial analysis and general practice data allows researchers and policy makers to identify chronic disease profiles at both the individual and community levels. This method of analysis further applies to the general practice level, where health care professionals in disease hot spots can increase the use of screening measures and related health education. Recognition of individuals and communities that require this increased surveillance would encourage the implementation of primary and secondary prevention techniques in general practices and related health services.
